# AGE/RAGE/DIAPH1 axis is associated with immunometabolic markers and risk of insulin resistance in subcutaneous but not omental adipose tissue in human obesity

**DOI:** 10.1038/s41366-021-00878-3

**Published:** 2021-06-08

**Authors:** Henry H. Ruiz, Anh Nguyen, Chan Wang, Linchen He, Huilin Li, Peter Hallowell, Coleen McNamara, Ann Marie Schmidt

**Affiliations:** 1 Diabetes Research Program, Division of Endocrinology, Diabetes and Metabolism, Department of Medicine, New York University Grossman School of Medicine, New York, NY USA; 2 Cardiovascular Division, Department of Medicine and Cardiovascular Research Center, University of Virginia, Charlottesville, VA USA; 3 Division of Biostatistics, Department of Population Health, New York University Grossman School of Medicine, New York, NY USA; 4 General Surgery Division, Department of Surgery, University of Virginia, Charlottesville, VA USA

**Keywords:** Obesity, Obesity, Translational research, Risk factors

## Abstract

**Background/objectives:**

The incidence of obesity continues to increase worldwide and while the underlying pathogenesis remains largely unknown, nutrient excess, manifested by “Westernization” of the diet and reduced physical activity have been proposed as key contributing factors. Western-style diets, in addition to higher caloric load, are characterized by excess of advanced glycation end products (AGEs), which have been linked to the pathophysiology of obesity and related cardiometabolic disorders. AGEs can be “trapped” in adipose tissue, even in the absence of diabetes, in part due to higher expression of the receptor for AGEs (RAGE) and/or decreased detoxification by the endogenous glyoxalase (GLO) system, where they may promote insulin resistance. It is unknown whether the expression levels of genes linked to the RAGE axis, including *AGER* (the gene encoding RAGE), Diaphanous 1 (*DIAPH1*), the cytoplasmic domain binding partner of RAGE that contributes to RAGE signaling, and *GLO1* are differentially regulated by the degree of obesity and/or how these relate to inflammatory and adipocyte markers and their metabolic consequences.

**Subjects/methods:**

We sought to answer this question by analyzing gene expression patterns of markers of the AGE/RAGE/DIAPH1 signaling axis in abdominal subcutaneous (SAT) and omental (OAT) adipose tissue from obese and morbidly obese subjects.

**Results:**

In SAT, but not OAT, expression of *AGER* was significantly correlated with that of *DIAPH1* (*n* = 16; $\hat \beta = 0.719$, [0.260, 1.177]; *q* = 0.008) and *GLO1* (*n* = 16; $\hat \beta = 0.773$, [0.364, 1.182]; *q* = 0.004). Furthermore, in SAT, but not OAT, regression analyses revealed that the expression pattern of genes in the AGE/RAGE/DIAPH1 axis is strongly and positively associated with that of inflammatory and adipogenic markers. Remarkably, particularly in SAT, not OAT, the expression of *AGER* positively and significantly correlated with HOMA-IR (*n* = 14; $\hat \beta = 0.794$, [0.338, 1.249]; *q* = 0.018).

**Conclusions:**

These observations suggest associations of the AGE/RAGE/DIAPH1 axis in the immunometabolic pathophysiology of obesity and insulin resistance, driven, at least in part, through expression and activity of this axis in SAT.

## Introduction

According to the World Health Organization (WHO), the incidence of obesity has nearly tripled over the past 45 years. While the precise cause of this phenomenon is not fully understood, increases in rates of obesity have been accompanied by nutrient excess, decreases in physical activity and a “westernization” of diet composition. In addition to higher fat and sugar content, Western-style diets are highly processed and represent a principal source of proteins non-enzymatically modified by sugars and/or lipids called advanced glycation end products (AGEs) [1], which have been linked to the pathophysiology of obesity and other cardiometabolic disorders [2]. There are multiple endogenous pre-AGE and AGE-detoxifying systems, principal among these is the glyoxalase (GLO) enzyme system, which converts the highly reactive glycating dicarbonyl methylglyoxal (MG) into the less toxic D-lactate [3]. Notably, recent observations suggest that excess MG due to inhibition or genetic deletion of glyoxalase 1 (*Glo1*) promotes obesity and impairs glucose metabolism and insulin resistance (IR) [4, 5]; likely by altering adipose tissue vascularization and promoting its expansion [6, 7]. Conversely, dietary manipulations to increase GLO1 activity improve vascular function and glycemic control in obese individuals [8].

When detoxification is incomplete or in settings of high AGE overload such as obesity and diabetes, AGEs exert their actions via multiple receptors but preferentially bind the receptor for advanced glycation end products (RAGE), which was first identified based on its ability to bind AGEs [9, 10]. RAGE is expressed in various tissues including adipose [11], lung, kidney, heart, epithelium and immune cells such as macrophages [12]. In addition to AGEs, the multi-ligand receptor RAGE also binds the S100/calgranulins, high mobility group protein box-1, amyloid β-peptide and oligomeric protein aggregates [13]. Based on its expression profile, its preferential ligands and the observations that one of the chief targets of its stimulation includes activation of the Nuclear Factor-κB complex [14, 15], RAGE has been primarily described as a mediator of inflammation. However, recent reports by our laboratory and others spotlight a role for RAGE in immunometabolism.

We previously reported that mice globally devoid of *Ager*; the gene encoding RAGE, wild-type (Wt) mice receiving bone marrow transplanted from mice devoid of *Ager*, or Wt mice treated with soluble RAGE, a splice isoform *of AGER* yielding a circulating protein which is hypothesized to act as a decoy receptor, all are protected from diet-induced obesity (DIO) and IR [16]. In parallel, Gaens et al. found that RAGE expression is significantly elevated in subcutaneous adipose tissue (SAT) from obese (body mass index [BMI] = 34.240 kg/m^2^) vs. lean (BMI = 23.440 kg/m^2^) non-diabetic individuals. Among severely obese subjects (BMI > 40 kg/m^2^), RAGE expression was higher in visceral than SAT [11]. The notion that RAGE in adipocytes plays a key role in obesity and IR was supported by our recent report that adipose-specific deletion of *Ager* protects mice from obesity and IR. In fact, surgical transplantation of adipose tissue devoid of brown or subcutaneous white adipocyte *Ager* into Wt mice is sufficient to endow these metabolic protections in high fat-fed mice [17].

Collectively, these reports highlight a crucial role for the AGE/RAGE axis in regulating obesity and IR, however, because RAGE lacks intracellular kinase signaling activity, the precise mechanism of action remains poorly understood. Our group discovered that Diaphanous 1 (DIAPH1), a member of the formin-family of Rho GTPase binding proteins with identified roles in cytokinesis, actin polymerization, cytoskeleton remodeling, and immune cell trafficking [18–20], binds to the intracellular domain of RAGE and contributes to RAGE signaling [21]. While it remains unknown whether or not DIAPH1 plays a central role in regulation of adipose tissue biology, obesity and IR, ongoing studies in our laboratory suggest that DIAPH1 is a key contributor to obesity. Taken together, current evidence suggests that the AGE/RAGE/DIAPH1 signaling axis is an important obesogenic pathway with multiple potential therapeutic targets.

Here, we sought to test the hypotheses that (1) *GLO1*, *AGER* and *DIAPH1* are differentially expressed in adipose tissue of adult non-diabetic/normoglycemic obese (Ob) and morbidly obese (MOb) human subjects, and (2) that the expression of these genes associates with that of prototypical markers of adipogenesis and adipose tissue inflammation, such as peroxisome proliferator-activated receptor gamma (PPARɣ) and Tumor necrosis factor alpha (TNFα). Furthermore, we assessed if such modulation is associated with clinically-relevant measures related to obesity. While absolute expression of genes in the AGE/RAGE/DIAPH1 axis in the surveyed adipose depots was unaltered by the degree of obesity, expression of genes in this axis strongly and positively correlated with markers of adipogenesis and inflammation, an effect that was more pronounced in SAT than OAT. Notably, we found that SAT, but not OAT *AGER* expression was a strong predictor of subjects’ homeostatic model assessment of insulin resistance (HOMA-IR). Thus, although the AGE/RAGE/DIAPH1 axis pattern of gene expression is not differentially affected by the degree of obesity, our findings support the notion that the degree of *AGER* expression associates with increased expression of adipogenic and inflammatory markers in SAT and clinical evidence of IR, further positioning RAGE as a crucial mediating gene in immunometabolism.

## Methods

### Subject samples

Subcutaneous and omental adipose tissues and blood were collected from sixteen (16) obese, non-diabetic human subjects prior to undergoing bariatric surgery. Summary of subjects’ anthropomorphic data is illustrated in Table 1. Subjects were grouped by BMI into obese (BMI < 40 kg/m^2^, *n* = 8) or MOb (BMI > 55 kg/m^2^, *n* = 8) and each group consisted of two males and six females. In other analyses, we considered body weight as a continuous variable for our correlation studies. Both groups were normoglycemic with no statistically-significant differences in fasting insulin or measures of IR including HOMA-IR [22] and the quantitative insulin sensitivity check index (QUICKI) [23]. Use of anti-hypertensive medications was comparable between groups. Informed consent was obtained from all subjects prior to sample collection and in complete compliance with, and strict adherence to, the guidelines approved by the University of Virginia (UVA) and New York University Langone Health’s institutional review boards.

**Table 1:** Subject anthropomorphic and clinical data.

.	Obese	Morbidly obese	*p* value
Age	44 (12.6) *n* = 8	42 (7.7) *n* = 8	0.655
Sex	2M/6F *n* = 8	2M/6F *n* = 8	N/A
Race	1B/7C *n* = 8	3B/5C *n* = 8	N/A
Height (in)	64.8 (4.4) *n* = 8	66.1 (4.7) *n* = 8	0.553
Weight (Lbs)	232.8 (36.9) *n* = 8	411.8 (41.4) *n* = 8	<0.0001**
BMI (Kg/m^2^)	38.8 (1.1) *n* = 8	66.6 (7.9) *n* = 8	<0.0001**
Hypertension	5Y/3N *n* = 8	5Y/3N *n* = 8	N/A
Beta-Blockers	1Y/7N *n* = 8	0Y/8N *n* = 8	N/A
Diabetes	0Y/8N *n* = 8	0Y/8N *n* = 8	N/A
A1C (%)	5.7 (0.5) *n* = 5	5.5 (0.3) *n* = 5	0.538
Fasting Glucose (mg/dL)	108 (35.5) *n* = 8	111 (35.8) *n* = 8	0.889
Fasting Insulin (µIU/ml)	11.4 (8.3) *n* = 8	20.5 (25.9) *n* = 6	0.365
HOMA-B (%)	88 (49.6) *n* = 7	148 (145.3) *n* = 6	0.325
HOMA-IR	3.5 (3.7) *n* = 8	6.6 (9.3) *n* = 6	0.405
QUICKI	0.15 (0.02) *n* = 8	0.14 (0.02) *n* = 6	0.514
Adiponectin (ng/ml)	6808 (2786) *n* = 8	4222 (719) *n* = 4	0.104
MCP1 (pg/ml)	220 (117) *n* = 8	226 (183) *n* = 4	0.944
Creatinine (mg/dL)	0.875 (0.10) *n* = 8	0.888 (0.16) *n* = 8	0.858
AST (U/L)	26 (7.5) *n* = 8	35 (33.5) *n* = 7	0.483
ALT (U/L)	34 (25.8) *n* = 8	43 (37.1) *n* = 8	0.603

Mean and standard deviation (SD) values for anthropomorphic variables, plasma clinical measures and calculated surrogate measures of insulin sensitivity.

**indicate statistically significant differences at *p*-value < 0.01.

### Clinical measures

Fasting blood glucose was measured using a glucometer (Accu-Chek Aviva). Circulating levels of fasting insulin, Hemoglobin A1c, adiponectin, Monocyte Chemoattractant Protein-1 (MCP-1), creatinine, aspartate transaminase (AST) and alanine transaminase (ALT) were measured at the clinical and research laboratories of UVA following established clinical protocols.

### Adipose tissue sample processing for protein/gene expression and AGE quantification

See supplemental methods.

#### Quantitative polymerase chain reaction (qPCR)

cDNA samples were diluted 1:10 in TaqMan fast universal master mix (Thermo Fisher Scientific, Cat. 4367846) containing a target gene (dye = FAM) and a housekeeping gene (dye = VIC). Samples were mixed, centrifuged and allowed to run for 40 cycles using a Fast 7500 real-time PCR system (Applied Biosystems). After adjusting curve thresholds, data were exported into excel files and the coefficient of variation (CV) was calculated for all duplicate measures. Samples with CV > 2.5% were identified and the curves were analyzed manually, selecting the data points with the most representative curve. Data were analyzed using the 2^^-(ddCt)^ method [24] normalized to housekeeping genes as controls. Each gene analysis was performed with its own housekeeping gene, the least variable housekeeping gene across samples was selected for analysis of the data presented. When comparing Ob vs. MOb samples, data were further normalized to the Ob group as reference. A list of the gene assay identification numbers is provided in supplemental Table 1.

#### Statistical analyses

Power calculations from experimental model data comparing AGE/RAGE/DIAPH1 axis gene changes in adipose tissue from lean and obese samples were used to determine the appropriate sample size. Anthropomorphic data were summarized as mean and standard deviation for continuous variables and count for binary variables. Normal data distribution was assessed and the appropriate nonparametric tests were used and reported when necessary. In normally distributed data, the *T*-test was employed to evaluate the comparison between Ob and MOb groups for continuous variables and the Fisher’s exact test was for binary variables. Linear models were conducted to investigate the correlations among the patterns of gene expression in subcutaneous and omental adipose tissues. For each target gene (*GLO1, AGER*, and *DIAPH1*), linear regressions were fitted to assess the association with transcript expression levels of the other genes. Samples where gene transcript was not detected were excluded from those analyses. Linear regressions were also used to determine whether gene expression patterns were associated with the renal, hepatic and adipose function as well as clinical measures of insulin sensitivity. Both age and sex were involved in all linear regressions for covariate adjustment. The Benjamini–Hochberg (BH) procedure [25] was applied to correct for multiple comparison testing. Beta hat ($\hat \beta$) is the estimated standardized coefficient based on the linear regression. The *q* value, a false discovery rate-adjusted *p* value, is calculated using the BH procedure for multiple testing correction. All statistical analyses were performed using the R software environment (Version 3.6) at an alpha level of *p* ≤ 0.05, selected *apriori* as the threshold for significance. *p*/*q* values > 0.05 and <0.100 were considered as statistical trends.

## Results

Analyses of subjects’ anthropomorphic data demonstrate that the Ob and MOb groups were clinically comparable with the exception of statistically-significant differences in body weight and BMI. Fasting blood glucose and insulin levels were similar between the two groups as was insulin sensitivity determined by HOMA-IR and QUICKI ratios. In line with these observations, HOMA-β, which is a surrogate measure of β-cell function [22], was not significantly different between groups. No differences between groups were observed in circulating levels of serum creatinine, AST, ALT, adiponectin and the chemoattractant protein, MCP-1 (Table 1).

**Fig. 1:**
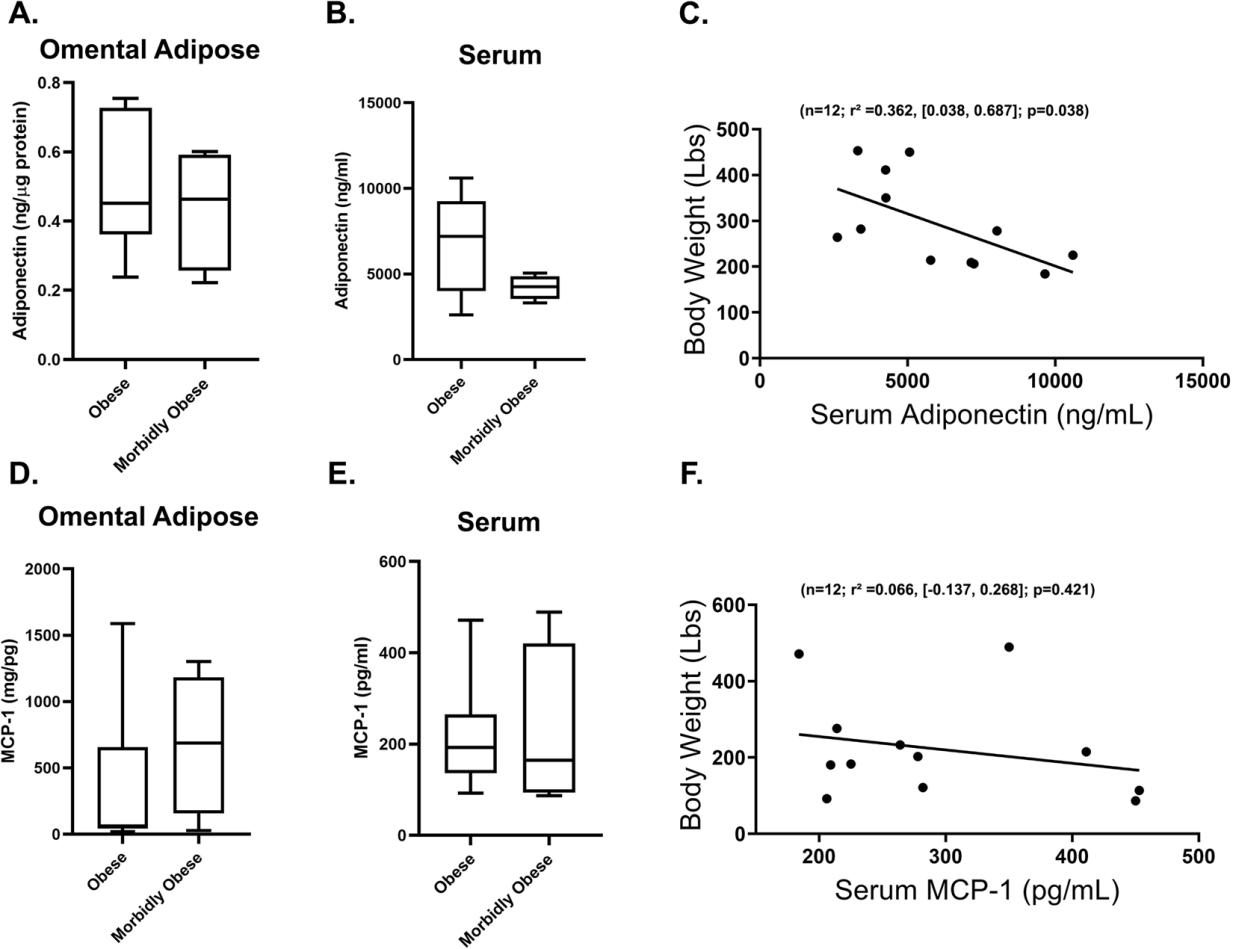
Circulating adiponectin but not MCP-1 correlate with body weight. **A** Omental adipose and (**B**) serum adiponectin levels in Ob vs. MOb subjects. **C** Regression plot depicting the association between body weight and serum adiponectin levels. **D** Omental adipose and (**E**) serum MCP-1 levels in Ob vs. MOb subjects. **F** Regression plot depicting the association between body weight and serum MCP-1. Box-and-whiskers plots illustrate within group minimum and maximum values range. For regression graphs, statistical significance and strength of association are indicated by *p* and *r*^2^ values respectively, 95% confidence interval in brackets, “n” indicates sample size.

Since no differences were observed in key markers of adipocyte function and systemic inflammation, we examined both SAT and OAT for these markers. Adiponectin content in OAT was not different between Ob and MOb subjects and while plasma adiponectin levels tended to be generally lower in the MOb vs. Ob groups (*p* = 0.104), this difference did not reach statistical significance (Fig. 1A–B). Although regression analysis showed no relationship between BMI and serum adiponectin levels, there was a statistically significant negative correlation between body weight and levels of serum adiponectin (*n* = 12; *r*^2^ = 0.362, [0.038, 0.687]; *p* = 0.038) (Fig. 1C). OAT and serum MCP1 levels did not differ between groups nor did serum MCP1 correlate with body weight (Fig. 1D–F).

**Fig. 2:**
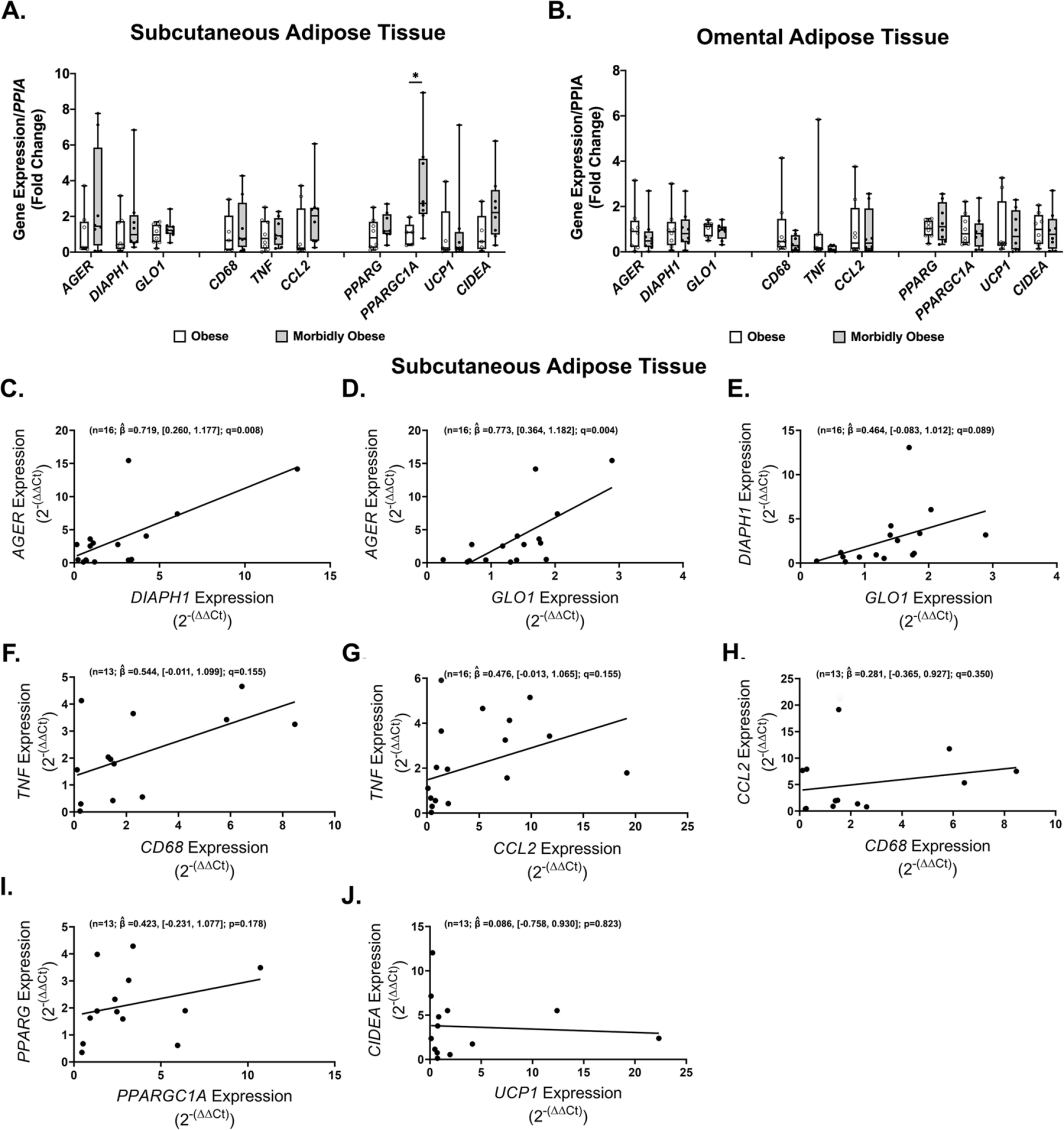
Significant association in expression pattern among genes in the AGE/RAGE/DIAPH1 axis in obese subcutaneous adipose tissue. Expression of target genes in MOb relative to Ob subjects in (**A**) subcutaneous and (**B**) omental adipose tissue. Regression plots for the association among (**C**–**E**) AGE/RAGE/DIAPH1 axis, prototypical (**F**–**H**) inflammatory and (**I**–**J**) adipose metabolism genes. Statistical significance and strength of association are indicated by *q* and $\hat \beta$ values respectively, 95% confidence interval in brackets, “n” indicates sample size.

To directly address the AGE/RAGE/DIAPH1 axis, we quantified the SAT and OAT expression of prototypical genes involved in this axis (*GLO1, AGER, DIAPH1)*, genes involved in adipose inflammation (*CD68, TNF, CCL2)* and adipocyte metabolism (*PPARG, PPARGC1A, UCP1, CIDEA)* to determine whether these are regulated or if they associate with clinically-relevant measures. With the exception of *PPARGC1A*, these genes were not differentially regulated in Ob vs. MOb subjects in SAT (Fig. 2A) or OAT (Fig. 2B). *PPARGC1*A expression was significantly higher in SAT but not OAT samples from MOb vs. Ob subjects (Fig. 2A).

Using data from all subjects, we probed whether gene expression patterns in each category associate with one another. *AGER* expression in SAT associated strongly and positively with *DIAPH1* (*n* = 16; $$\hat \beta$$$$= 0.719$$, [0.0.260, 1.177]; *q* = 0.008) and *GLO1* (*n* = 16; $$\hat \beta = 0.773$$, [0.364, 1.182]; *q* = 0.004) while a statistical trend (*n* = 16; $$\hat \beta$$$$= 0.464$$, [−0.083, 1.012]; *q* = 0.089) in the same direction was observed for the association between *DIAPH1* and *GLO1* (Fig. 2C–E). No statistically significant correlations were observed between genes associated with inflammation or adipocyte metabolism (*CD68, TNF, CCL2, PPARGC1A, and CIDEA)* (Fig. 2F–J) in SAT. Remarkably, in contrast to SAT, in OAT samples, genes in the AGE/RAGE/DIAPH1 axis did not correlate with one another (Supplemental Fig. 1A-C), while statistically significant associations were observed between *CD68*, *TNF* and *CCL2* (Supplemental Fig. 1D–F) and *PPARG* with *PPARGC1A* (*n* = 16; $$\hat \beta = 0.500$$, [0.124, 0.876]; *p* = 0.013) but not between *UCP1* and *CIDEA* (Supplemental Fig. 1G–H). Taken together, these data suggest a potential dichotomy whereby expression of genes in the AGE/RAGE/DIAPH1 axis is closely associated with each other in SAT but not OAT in obesity, while inflammatory genes associate with each other in OAT but not SAT.

**Fig. 3:**
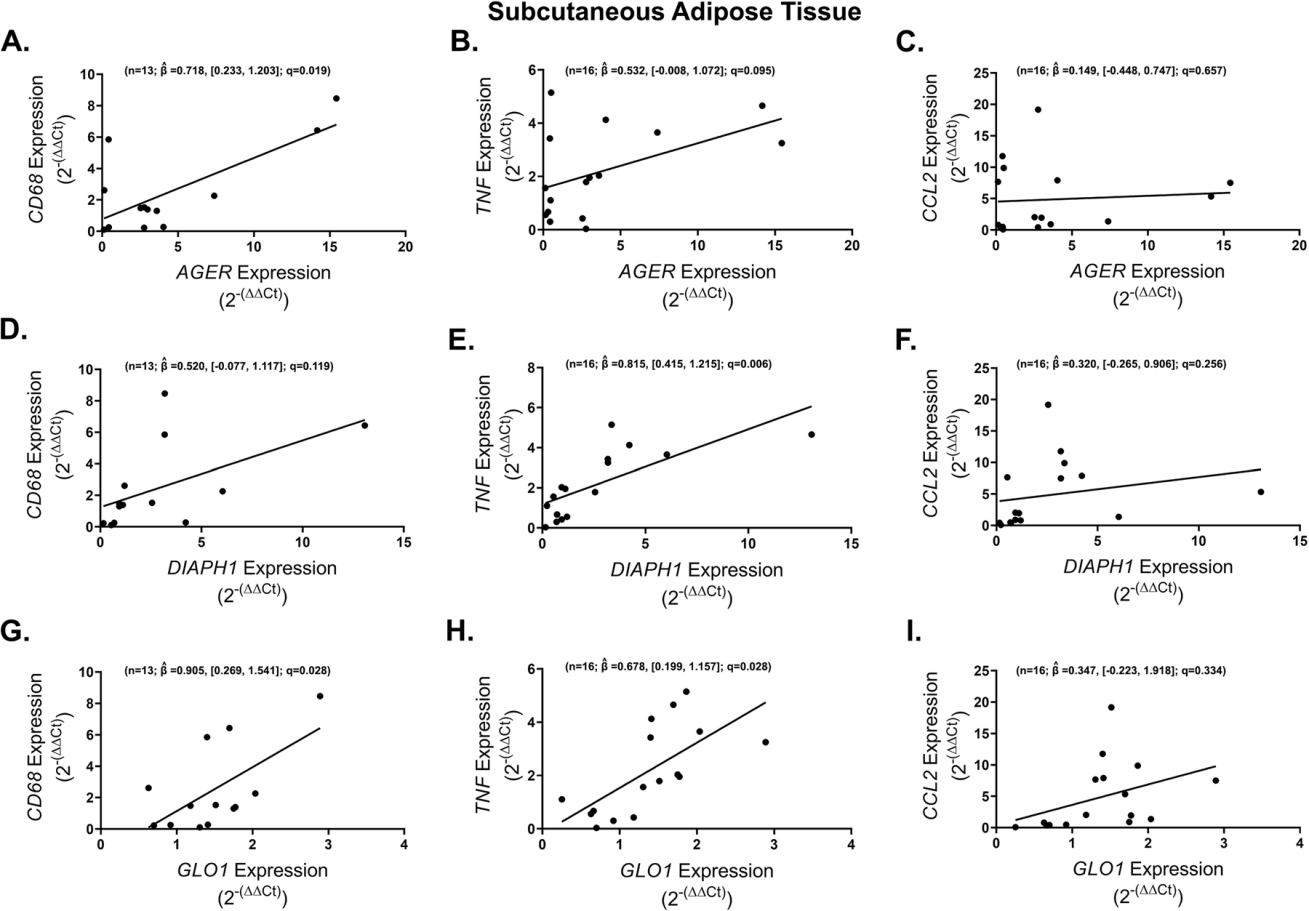
Expression of obese subcutaneous adipose tissue AGE/RAGE/DIAPH1 axis genes positively correlates with prototypical inflammatory markers. Regression plots depicting the association in subcutaneous adipose tissue between *CD68*, *TNF* and *CCL2* with (**A**–**C**) *AGER*, (**D**–**F**) *DIAPH1* and (**G**–**I**) *GLO1* respectively. Statistical significance and strength of association are indicated by *q* and $\hat \beta$ values respectively, 95% confidence interval in brackets, “n” indicates sample size.

We next determined whether genes in the AGE/RAGE/DIAPH1 axis associate with markers of adipose tissue inflammation. SAT *AGER* expression was strongly and positively correlated with *CD68* (*n* = 13; $\hat \beta = 0.718$, [0.233, 1.203]; *q* = 0.019) and tended (*n* = 16; $\hat \beta = 0.532$, [−0.008, 1.072]; *q* = 0.095) to correlate in the same direction with *TNF* while no association was observed with *CCL2* (Fig. 3A–C). Comparable to that of *AGER*, in SAT, *DIAPH1* levels significantly and positively correlated with *TNF* (*n* = 16; $\hat \beta = 0.815$, [0.415, 1.215]; *q* = 0.006) expression but not with *CD68* or *CCL2* (Fig. 3D–F) and increased SAT *GLO1* expression was significantly associated with higher levels of *CD68* (*n* = 13; $\hat \beta = 0.905$, [0.269, 1.541]; *q* = 0.028) and *TNF* (*n* = 16; $\hat \beta = 0.678$, [0.199, 1.157]; *q* = 0.028) but not CCL2 (Fig. 3G–I).

We tested for these associations in OAT and found that other than a statistical trend (*n* = 16; $\hat \beta = 0.646$, [0.030, 1.263]; *q* = 0.083) for higher expression of *CD68* with increasing levels of *GLO1*, no significant correlations were observed between *AGER* and *DIAPH1* gene sets (Supplemental Fig. 2). Together, these observations suggest a close association between the AGE/RAGE/DIAPH1 axis in SAT but not in OAT inflammation.

**Fig. 4:**
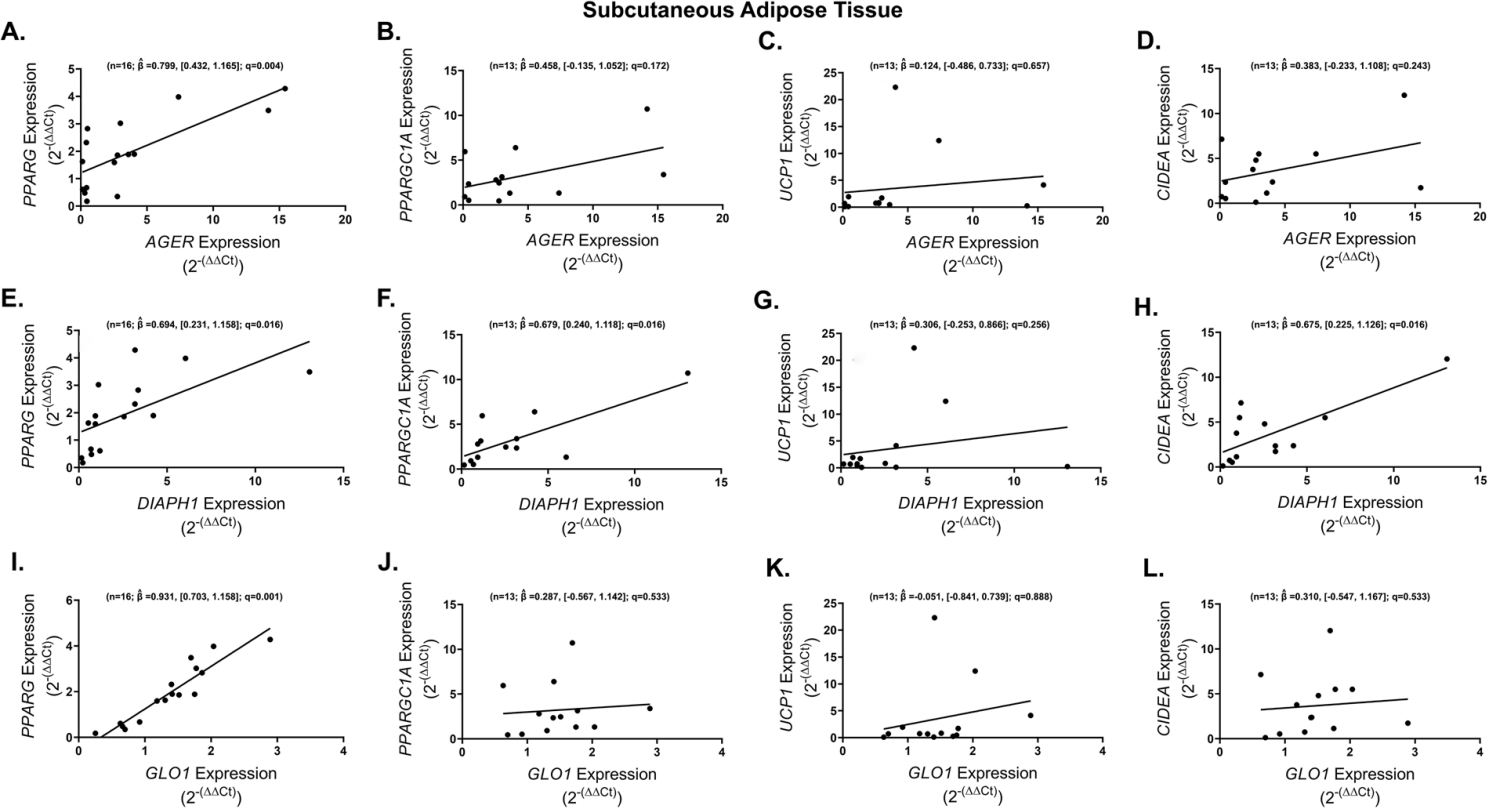
Obese subcutaneous adipose tissue PPARG expression is closely associated with the AGE/RAGE/DIAPH1 axis genes. Regression plots depicting the association in subcutaneous adipose tissue between *PPARG, PPARGC1A, UCP1* and *CIDEA* with (**A**–**D**) *AGER*, (**E**–**H**) *DIAPH1* and (**I**–**L**) *GLO1* respectively. Statistical significance and strength of association are indicated by *q* and $\hat \beta$ values respectively, 95% confidence interval in brackets, “n” indicates sample size.

We also sought to determine if the AGE/RAGE/DIAPH1 axis is linked to markers of adipogenesis (*PPARG* & *PPARGC1A*) and metabolic genes that we previously identified to be regulated by RAGE in a mouse model of obesity (*UCP1* and *CIDEA*) [17]. SAT *AGER* was found to significantly and positively correlate with expression of *PPARG* (*n* = 16; $\hat \beta = 0.799$, [0.432, 1.165]; *q* = 0.004) while no association was observed with *PPARGC1A, UCP1* and CIDEA (Fig. 4A–D). SAT *DIAPH1* exhibited significant and positive associations with *PPARG* (*n* = 16; $\hat \beta = 0.694$, [0.231, 1.158]; *q* = 0.016), *PPARGC1A* (*n* = 13; $\hat \beta = 0.679$, [0.240, 1.118]; *q* = 0.016) and *CIDEA* (*n* = 13; $\hat \beta = 0.675$, [0.225, 1.126]; *q* = 0.016) but not with *UCP1* (Fig. 4E–H). Similar to *AGER*, higher expression of SAT *GLO1* was significantly associated with higher expression of *PPARG* (*n* = 16; $\hat \beta = 0.931$, [0.703, 1.158]; *q* = 0.001) but not the other genes probed (Fig. 4I–L). In OAT samples, *GLO1* expression levels, but not *AGER* or *DIAPH1*, were significantly and positively correlated with *PPARGC1A* (*n* = 16; $\hat \beta = 0.988$, [0.586, 1.391]; *q* = 0.001) and *CIDEA* (*n* = 16; $\hat \beta = 0.819$, [0.380, 1.258]; *q* = 0.006). No other statistically significant correlations were observed for these comparisons in OAT (Supplemental Fig. 3). Thus, as with inflammatory markers, the AGE/RAGE/DIAPH1 axis strongly associates with markers of adipocyte metabolism in SAT but not in OAT, with the exception of *GLO1* and its relationship to *PPARGC1A* in OAT.

**Fig. 5:**
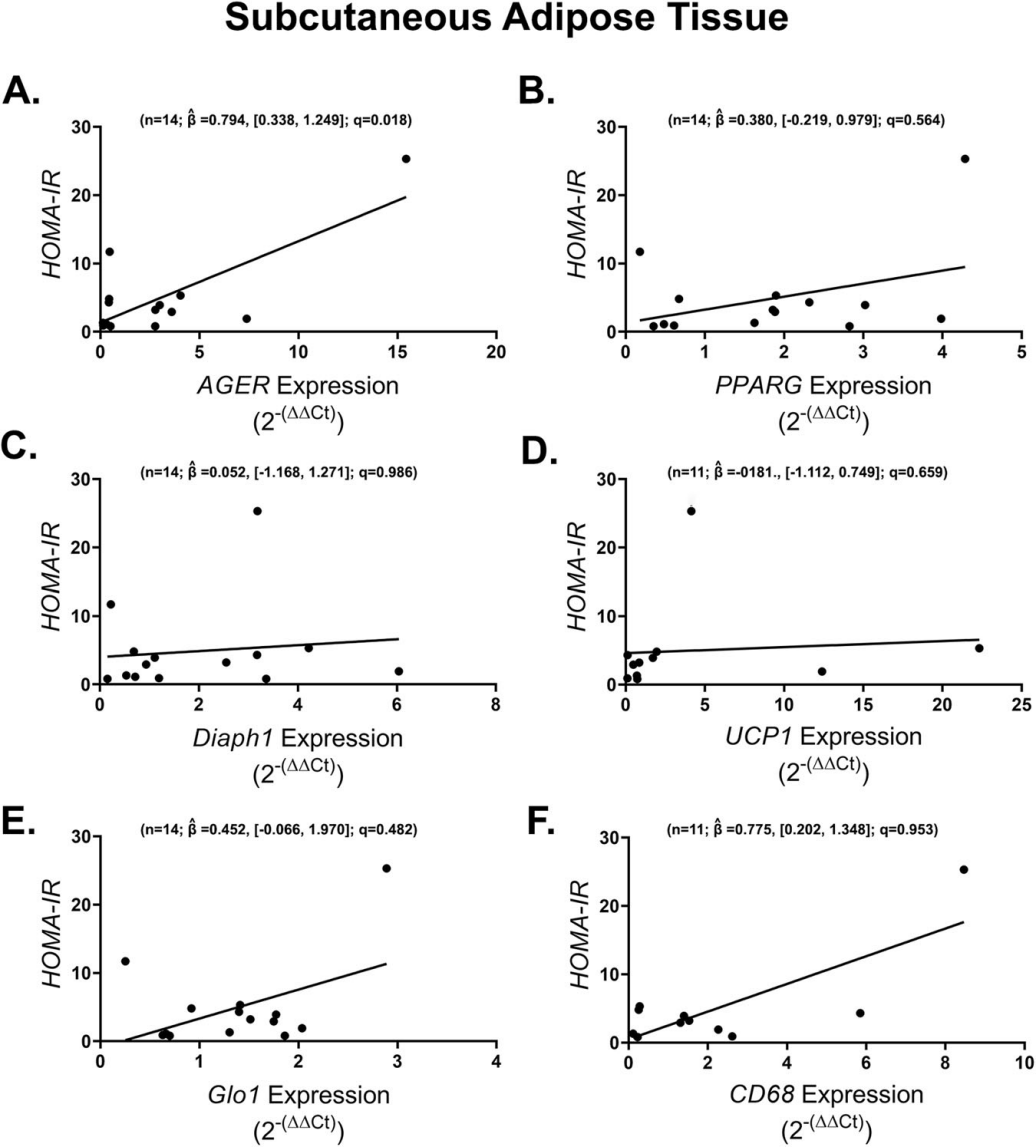
Obese subcutaneous adipose tissue *AGER* expression is positively associated with HOMA-IR. Regression plots depicting the association in subcutaneous adipose tissue between (**A**) *AGER*, (**B**) *PPARG*, (**C**) *DIAPH1*, (**D**) *UCP1*, (**E**) *GLO1*, (**F**) *CD68* and subjects’ HOMA-IR. Statistical significance and strength of association are indicated by *q* and $\hat \beta$ values respectively, 95% confidence interval in brackets, “n” indicates sample size.

We then assessed whether or not the expression of selected genes in the AGE/RAGE/DIAPH1 axis, inflammatory or adipose metabolism categories had predictive power for the various clinically-relevant parameters measured in this study. In SAT, only *AGER* expression, but not that of *PPARG, DIAPH1, UCP1, GLO1* or *CD68*, was found to be significantly and positively correlated with HOMA-IR (*n* = 14; $\hat \beta = 0.794$, [0.338, 1.249]; *q* = 0.018) (Fig. 5). In contrast, in OAT, there were no associations between any of these AGE/RAGE/DIAPH1, adipogenic or inflammatory factors with HOMA-IR (Supplement table 3).

Finally, we asked whether there were associations with genes of the AGE/RAGE/DIAPH1 axis and other immunometabolic genes with the clinical measures assessed in this study. After correction for multiple statistical tests, no other statistically significant gene/clinically-relevant-measure correlations were observed in SAT (Supplemental Table 2) or OAT (Supplemental Table 3). Thus, in these adipose depots, of the genes examined in this study, higher expression of SAT *AGER* was found to be linked to higher HOMA-IR values in non-diabetic obese subjects.

To ascertain whether the associations we have uncovered between genes in the AGE/RAGE/DIAPH1 axis and inflammatory markers as well as clinical measures of IR in SAT might be explained by differential AGE burden in the two adipose depots, we measured AGEs content by two distinct methods: first, from acid hydrolyzed fraction extraction and second, via immunoblotting. We found that AGE content per protein of adipose tissue is comparable between SAT and OAT of our study subjects (Supplemental Fig. 4A). There were no statistically significant differences in AGE content in either SAT (Supplemental Fig. 4B) or OAT (Supplemental Fig. 4C) when comparing obese to MOb subjects. These observations were confirmed by immunoblotting studies (Supplemental Fig. 4D–E). Furthermore, regression analyses indicated that adipose tissue AGE content is not correlated with the expression of genes in the AGE/RAGE/DIAPH1 axis in SAT (Supplemental Fig. 5A–C) or OAT (Supplemental Fig. 5D–F). Thus, differences in AGE content between SAT and OAT do not explain our reported associations between genes in the AGE/RAGE/DIAPH1 axis, inflammatory markers and HOMA-IR.

## Discussion

There is growing experimental evidence supporting a link between AGE/RAGE/DIAPH1 and the pathophysiology of obesity and associated metabolic disorders. However, whether these observations are relevant to obesity in human subjects has yet to be fully established. When compared to lean subjects, obese individuals exhibit lower levels of circulating AGEs in parallel with higher adipose tissue expression of RAGE and greater tissue accumulation of AGEs [26], which has led to the tissue “AGE trapping” hypothesis, whereby obesity is characterized by excess tissue AGE buildup and, consequently, low circulating AGEs [26]. Notably, these increases in adipose tissue AGEs are associated with greater incidence of IR and risk for metabolic dysfunction. If the expression of *GLO1/RAGE/DIAPH1* in adipose tissue is altered by increasing degrees of obesity and whether the expression of these genes associates with adipose tissue inflammation and adipocyte metabolism markers was addressed in the present studies.

To avoid the potential confounding effects of hyperglycemia on AGEs and RAGE expression, we studied non-diabetic subjects who differed significantly in their degree of obesity. With the exception of body weight and BMI, study subjects had comparable clinical history, anthropomorphic and IR measures. Furthermore, levels of surrogate measures for renal and hepatic function, and systemic inflammation were similar between the two groups. There were no differences in either omental adipose or circulating adiponectin levels between groups while serum adiponectin levels negatively associated with body weight. Using two complementary approaches we found no statistically significant differences in AGE burden between adipose depots nor between obese and MOb subjects in either adipose depot. AGE burden was not associated with differences in *AGER, DIAPH1 or Glo1* and thus we conclude that adipose tissue AGEs content does not confound the associations we identified between the AGE/RAGE/DIAPH1 axis and metabolic variables.

Our first hypothesis was that compared to obese subjects, *AGER*/*DIAPH1* expression in adipose tissue would be significantly higher in subjects with morbid obesity whereas lower expression of mRNA transcripts encoding the AGE-detoxifier enzyme *GLO1* was predicted in those individuals. We did not observe statistically-significant differences in the expression of these genes between groups in either SAT or OAT. However, there appeared to be a tendency for greater *AGER* expression in SAT from MOb subjects, an effect for which statistical significance might have been obscured by our limited sample size. Furthermore, we speculated that transcript levels for *PPARG, PPARGC1A, CD68*, *TNFα* and *CCL2* would be higher in MOb subjects consistent with greater adiposity and inflammation. We found *PPARGC1A* expression to be significantly higher in SAT of MOb vs. obese subjects, however, none of the other target genes were differentially expressed in either adipose depot studied nor did the plasma levels of the protein encoded by *CCL2* (MCP-1) differ in OAT. Thus, we conclude that with the exception of the gene encoding PGC1α, genes in the AGE/AGER/DIAPH1 axis, inflammatory and adipocyte metabolism markers are not differentially regulated by the degree of obesity among obese subjects. Whether the expression of these genes differs between the lean state, mild and/or extreme obesity remains an open question.

Our second hypothesis was that *GLO1*/*AGER*/*DIAPH1* expression would correlate with one another and positively associate with the expression of inflammation and adipocyte metabolism markers in SAT and OAT. Regression analyses of the association of these genes in SAT revealed several novel correlations. In line with their known close interactions, the expression of *GLO1, AGER* and *DIAPH1* strongly associated with each other. Specifically, subjects expressing higher *AGER* also expressed higher levels of *DIAPH1* and *GLO1* in SAT. Notably, subjects with greater SAT *AGER* exhibited higher HOMA-IR ratios suggestive of a potential link between SAT *AGER* and IR even in clinically non-diabetic patients. We did not observe any association amongst these genes in OAT nor with clinically-relevant measures of IR.

PPARɣ and PGC1α play key roles in adipogenesis and insulin sensitization, and reportedly increase in expression during obesity [27, 28]. Further, PPARɣ expression is negatively correlated with cardiovascular risks [29] and IR [30]. We did not observe differences between obese and MOb subjects in the expression levels for these genes in either SAT or OAT; these subjects also did not differ by measures of insulin sensitivity. Statistical regression analyses, however, revealed strong and positive associations between all three genes in the AGE/RAGE/DIAPH1 axis and *PPARG* expression, which may indicate a potential counter-regulatory mechanism to defend from the negative effects of higher *AGER* and *DIAPH1* expression. The veracity of this hypothesis, which remains to be fully tested, is strengthened by the observation that in SAT, only *AGER* expression was predictive of HOMA-IR. Of note, these correlations were specific for *AGER* and *DIAPH1* expression in SAT as these associations were not observed in OAT. While increases in the PPARɣ axis have been associated with improved insulin sensitivity, the fact that neither *PPARG* nor *PPARGC1A* correlated with HOMA-IR suggest that in severe obesity, the observed increases are instead, likely a reflection of greater adipogenesis where *AGER* has also been implicated and may explain the strong correlation between these genes. Thus, we speculate that the association between SAT *AGER* and HOMA-IR are likely independent of the PPAR axis.

On account of the lack of mechanistic link between *AGER* and *PPAR* and expected changes associated with IR, we examined prototypical markers of inflammation. *CD68* is a prototypical marker for adipose tissue macrophages, which contribute to secretion of TNF. *TNF* is also expressed by adipocytes; its expression is higher in adipose tissue from obese vs. lean humans, and high levels are associated with hyperinsulinemia and IR [31, 32]. We did not observe significant differences between obese and MOb subjects in either SAT or OAT with regards to *CD68* and *TNF* expression. While this was unexpected because increasing obesity is commonly associated with OAT inflammation, it may be explained by previous observations that adipose *TNF* levels are less variable in subjects with BMIs >40, as is the case in our samples [31]. Interestingly, statistical regression analyses revealed correlations between the AGE/RAGE/DIAPH1 axis genes and the inflammatory markers *CD68* and *TNF* in SAT, but not OAT. This association appears to be in conflict with the established notion that OAT dysfunction has a superior contribution to whole-body metabolic impairment than other adipose depots including SAT [33–35]. This *status quo* has largely been skewed by studies specifically focusing on OAT analyses [36] and by comparisons of lean vs. obese/diabetic subjects [37]. Whether this is the case amongst obese human subjects is not as clear cut. In fact, there is a growing body of evidence that supports the argument that in obesity, SAT dysfunction (e.g., inflammation [38, 39], senescence [40] and morphological organization [41]) is strongly associated with metabolic disruptions including IR in humans. Thus, our finding that among obese subjects, the AGE/RAGE/DIAPH1 axis in SAT but not OAT associates with inflammation and IR is not unfounded and opens the possibility for an adipose-depot specific differential role for this axis in humans. Furthermore, using an experimental model of obesity, we recently highlighted a primary role for SAT RAGE in promoting obesity [17]. Specifically, transplantation of SAT devoid of adipocyte *Ager* was sufficient to prevent obesity and IR in Wt mice fed a high fat diet. Thus, the novel finding that *AGER* associated with human SAT but not OAT inflammation and IR is in line with our findings using rodent models. Altogether, these observations support and add to a repertoire of studies implicating RAGE as a key immunometabolic player in human obesity and open the possibility that common transcription factors may be regulating the genes in the AGE/RAGE/DIAPH1 axis, inflammation and adipose metabolism. Future studies will test these concepts. Importantly, *CCL2* expression did not associate with any genes in the AGE/RAGE/DIAPH1 axis in either tissue and OAT expression levels of the protein it encodes, MCP1, were not different between the two groups, suggesting that MCP1 responses in obesity are likely AGE/RAGE/DIAPH1-independent and suggest novel mechanism(s) by which RAGE is associated with increased macrophage markers (such as CD68 and *TNF*) in human and mouse obesity.

**Fig. 6:**
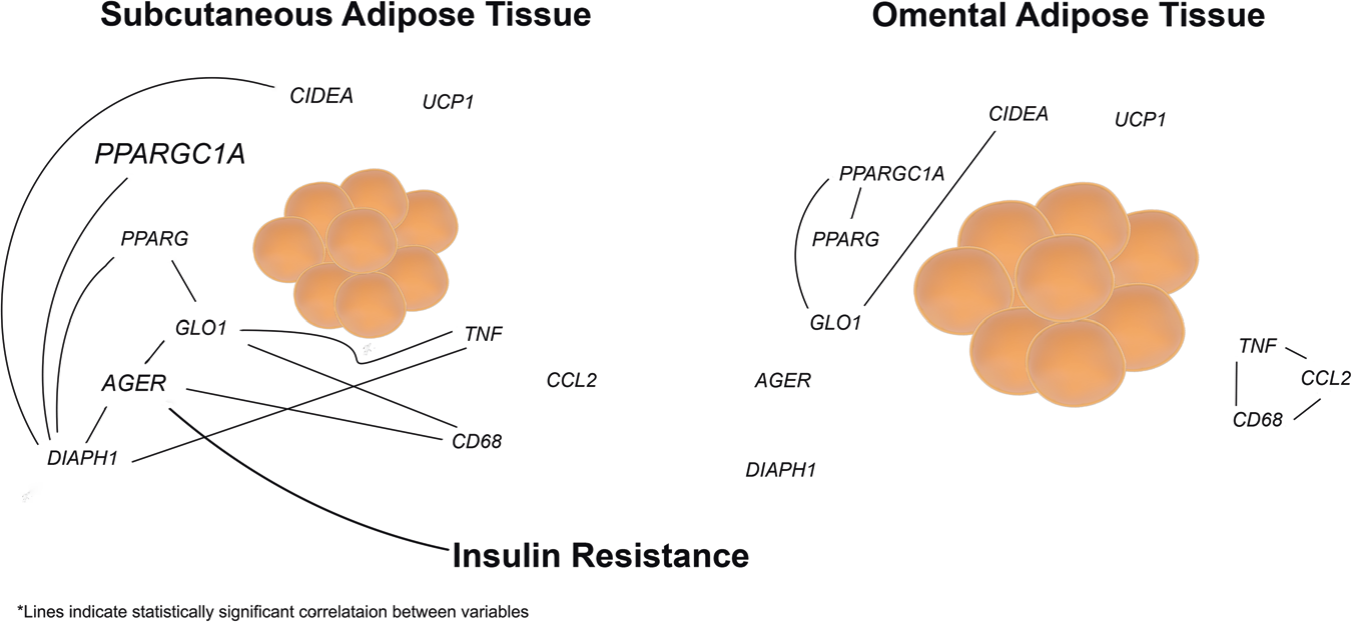
Proposed model of the AGE/RAGE/DIAPH1 axis in obesity. We propose that our data define a novel paradigm in which SAT *AGER* is significantly associated with insulin resistance, at least in part through perturbation of seminal immune and metabolic pathways.

While our observations provide a set of novel associations in SAT between the AGE/RAGE/DIAPH1 axis and adipose tissue inflammation and metabolism, there are notable limitations to our study. First, due to the strict selection criteria to obtain samples representative of significantly different degrees of obesity, our sample size may have prevented us from unveiling more key associations due to lack of statistical power. Second, the limited availability of tissue only allowed us to assess the expression of a select number of genes and whether or not the observed changes in gene expression translate into changes at the protein level remains to be determined in future studies. Further, in this study, although we were unable to address the questions raised by our hypothesis using alternative approaches such as flow cytometry and/or immunohistochemistry, this work sets the stage for future experimental and clinical studies in which tissues may be employed for these types of analyses. Such studies are particularly necessary to determine whether the association between AGEs/RAGE/Diaph1 and IR in humans can be attributed to a cause-and-effect relationship. Finally, because adipose tissue samples from lean individuals undergoing surgical procedures are difficult to obtain in a controlled manner, our conclusions are limited to comparisons amongst obese vs. MOb subjects, thus leaving open the question as to how the genes studied here compare between lean and obese subjects. Nevertheless, our novel observations highlight a role for the AGE/RAGE/DIAPH1 axis in the pathophysiology of obesity and IR. Remarkably, these findings pinpoint a new paradigm of RAGE-dependent immunometabolic regulation in SAT, not OAT, that likely is not directly dependent on PPAR- or TNF-driven mechanisms (Fig. 6). Indeed, as blockade of TNF has not shown consistent results in regulation of IR in human obesity [42–44], our findings may suggest probing potential roles for RAGE as a novel target for therapeutic intervention in obesity and its immunometabolic complications.

## Supplementary information

Supplemental Methods and Figure Legends

Supplemental Figure 1

Supplemental Figure 2

Supplemental Figure 3

Supplemental Figure 4

Supplemental Figure 5

Supplemental Table 1

Supplemental Table 2

Supplemental Table 3
