# Supplementary material for: AGE/RAGE/DIAPH1 axis is associated with immunometabolic markers and risk of insulin resistance in subcutaneous but not omental adipose tissue in human obesity
Source: Int J Obes (Lond). 2021 Jun 8;45(9):2083–94. doi: 10.1038/s41366-021-00878-3 (PMC8380543; doi:10.1038/s41366-021-00878-3)
Supplement: Supplementary file 1 — Supplemental Methods and Figure Legends [file 41366_2021_878_MOESM1_ESM.docx]

**Ruiz et al. AGE/RAGE/DIAPH1 axis is associated with immunometabolic markers and risk of insulin resistance in subcutaneous but not omental adipose tissue in human obesity.**

**Supplemental Methods**

**Adipose Tissue Protein/AGE Extraction/Quantification**

Approximately 1 gram of adipose tissue was homogenized in 1 mL of RIPA buffer (1% NP-40, 1% deoxycholate, 0.1% SDS, 150 mM NaCl, 50 mM Tris-HCL pH 7.6, 1 mM EDTA) containing 10 µL/mL protease inhibitors (Sigma; Cat # P8340)). Protein extracts were subjected to centrifugation at 4°C (3900X g force, X 10 minutes). Soluble homogenate was isolated and sample protein content quantified using a Pierce BCA Protein Assay Kit (Thermo Fisher Scientific). Adiponectin and MCP-1 content was assessed by ELISA (ReadySetGo ELISA, eBiosciences) following the manufacturer’s protocol.

Adipose Tissue RNA Extraction/cDNA Synthesis

Approximately 200 mg of abdominal subcutaneous or omental adipose tissue was immersed in 750µL of ice-cold QIAzol lysis reagent (Qiagen, Cat. 79306) and lyzed at 4°C using a handheld tissue homogenizer (Qiagen, Cat. 9002755) with disposable probes (Qiagen, 990890) for four 15 seconds (s) bouts with 10 s intermissions. RNA was then extracted from the homogenates using an RNeasy lipid tissue kit (Qiagen, Cat. 74804) following the manufacturer’s instructions. RNA content and quality (all 260/280 ratios >1.9, <2.1) were assessed in 1µL volume using a NanoDrop ND-1000 UV-Vis Spectrophotometer (Thermo Fisher Scientific). All samples were diluted to 1µg RNA followed by cDNA synthesis using an iScript kit (Bio-Rad, Cat. 1708891). cDNA samples were stored at -20°C until analyzed.

AGEs Extraction/Quantification from Adipose Tissue

On average, 130mg of SAT or OAT samples were homogenized in 300µL complete RIPA buffer in reinforced 1mL tubes containing 2.8mm sterile zirconium ceramic oxide beads (Fisher Scientific, Cat. 15-340-160) using a bead mill homogenizer (Omni International, Cat. 19-050A). Tissue homogenates were divided into two aliquots of 90µL and 210µL for acid hydrolysis extraction of AGEs and immunoblotting respectively. 90µL of SAT, OAT and RIPA (blank control) samples were mixed with 90µL of ice cold 1N trichloroacetic acid (TCA) and incubated at 4°C for 20 minutes. Samples were then centrifuged at 15,000rpm for 10 minutes and the resulting pellets were washed three times in 500 µL of ice-cold ether. Pellets were transferred to borosilicate glass tubes (Fischer Scientific, Cat. 14-932-1C), resuspended in 2mL of 6N deoxygenated hydrochloric acid, blanketed with gas nitrogen and heated at 110°C for 18 hours. Samples were transferred to 2mL tubes and vacuum dried overnight. Samples were then reconstituted in 1mL of distilled water and loaded in technical triplicates in a 96-well plate for fluorescence detection in a spectrofluorometer (Beckman Coulter, DTX 880) at 370/440nm excitation/emission wavelengths. Averaged fluorescence readout from blank samples was subtracted from all wells and then AGEs fluorescence recorded.

For immunoblotting studies, 210µL of SAT and OAT lysates were centrifuged at 13,000g for 15 minutes at 4°C and the intranatant was collected. Samples were diluted to 1µg/µL and mixed with sample reducing agent and loading dye (Thermo Scientific, Waltham, MA). 15 µg of protein were separated in 4-20% Midi-PROTEAN TGX gels (Bio-Rad, Hercules, CA) and blotted onto nitrocellulose membranes (Bio-Rad, Hercules, CA). Membranes were blocked for 1 hour with Odyssey LI-COR Blocking Buffer (LI-COR, Lincoln, NE) at room temperature and incubated with anti-AGE antibody (Abcam, Cat. ab23722) overnight at 4°C. Membranes were washed 3 times for at least 5 mins each in TBS-T (0.1%) and incubated with IRDye 800CW goat anti–rabbit IgG or IRDye 680RD goat anti–mouse IgG (LI-COR, Lincoln, NE) for 1 hour at room temperature in blocking buffer containing 0.1% TBS-T and 0.1% SDS. Membranes were washed 3 times in TBS-T followed by a final wash in TBS, scanned with the LI-COR Odyssey Classic (LI-COR, Lincoln, NE). Antibodies were stripped by incubating membranes in a 0.2M sodium hydroxide solution, washed in TBS/T and blocked as above. Membranes were then probed with an anti-β actin antibody (Santa Cruz, Cat. sc-47778). AGEs (whole lane) and β actin bands were quantified with Image Studio Lite software (LI-COR, Lincoln, NE) based on direct fluorescence measurement and AGEs signal was further normalized to actin signal.

**Supplemental Figure Legends**

**Supplemental Figure 1. Expression of inflammatory but not metabolic or AGE/RAGE/DIAPH1 axis genes associate in obese omental adipose tissue.** Regression plots representing the association in omental adipose expression of genes in the AGE/RAGE/DIAPH1 axis: *AGER* with A) *DIAPH1* and B) *GLO1*, and between C) *DIAPH1* and *GLO1*; among inflammatory markers: *TNF* with D) *CD68* and E) *CCL2*, and between F) *CD68* and *CCL2*; and among metabolic markers: G) *PPARG* and *PPARGC1A*, and H) *UCP1* and *CIDEA*. Statistical significance and strength of association are indicated by q and
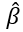
values respectively, 95% confidence interval in brackets, “n” indicates sample size.

**Supplemental Figure 2. Omental adipose tissue expression of AGE/RAGE/DIAPH1 genes does not correlate with inflammatory markers.** Regression plot representing the association in omental adipose tissue between *CD68*, *TNF* and *CCL2* with A-C) *AGER*, D-F) *DIAPH1* and G-I) *GLO1* respectively. Statistical significance and strength of association are indicated by q and
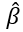
values respectively, 95% confidence interval in brackets, “n” indicates sample size.

**Supplemental Figure 3. *GLO1* expression in obese omental adipose is positively correlated with the expression of *PPARGC1A* and *CIDEA*.** Regression plots depicting the association in omental adipose tissue between *PPARG, PPARGC1A, UCP1* and *CIDEA* with A-D) *AGER*, E-H) *DIAPH1* and I-L) *GLO1* respectively. Statistical significance and strength of association are indicated by q and
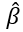
values respectively, 95% confidence interval in brackets, “n” indicates sample size.

**Supplemental Figure 4. AGE content is comparable between SAT and OAT depots and between obese and morbidly obese human subjects.** TCA hydrolyzed fraction AGE levels normalized to protein content in A) OAT and SAT and adipose tissue AGE content comparison between obese and morbidly obese subjects in B) SAT and C) OAT. Western blot detection of AGEs and β-actin in protein lysates from SAT D) and OAT E) depots with the respective fluorescence quantification. Data presented as individual data points with means ± standard error of the mean (SEM).

**Supplemental Figure 5. AGEs content in SAT and OAT do not correlate with the expression of genes in the AGE/RAGE/DIAPH1 axis.** Regression plot representing the association in SAT (1-C) and OAT (D-F) between tissue AGEs content and the expression of *AGER*, *DIAPH1* and *GLO1* respectively. Statistical significance and strength of association are indicated by q and
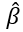
values respectively, 95% confidence interval in brackets, “n” indicates sample size.

**Supplemental Table 1. Gene target list.** List of all genes assessed in this study and the TaqMan assay identification number.

**Supplemental Table 2. Regression analysis table for associations between target genes and clinical measures in subcutaneous adipose tissue.** Correlation between target genes and clinical outcomes including A1C%, ALT, AST, creatinine, HOMA-IR and QUICKI.

**Supplemental Table 3. Regression analysis table for associations between target genes and clinical measures in omental adipose tissue.** Correlation between target genes and clinical outcomes including A1C%, ALT, AST, creatinine, HOMA-IR and QUICKI.
