# Supplementary figures and images for: AGE/RAGE/DIAPH1 axis is associated with immunometabolic markers and risk of insulin resistance in subcutaneous but not omental adipose tissue in human obesity
Source: Int J Obes (Lond). 2021 Jun 8;45(9):2083–94. doi: 10.1038/s41366-021-00878-3 (PMC8380543; doi:10.1038/s41366-021-00878-3)

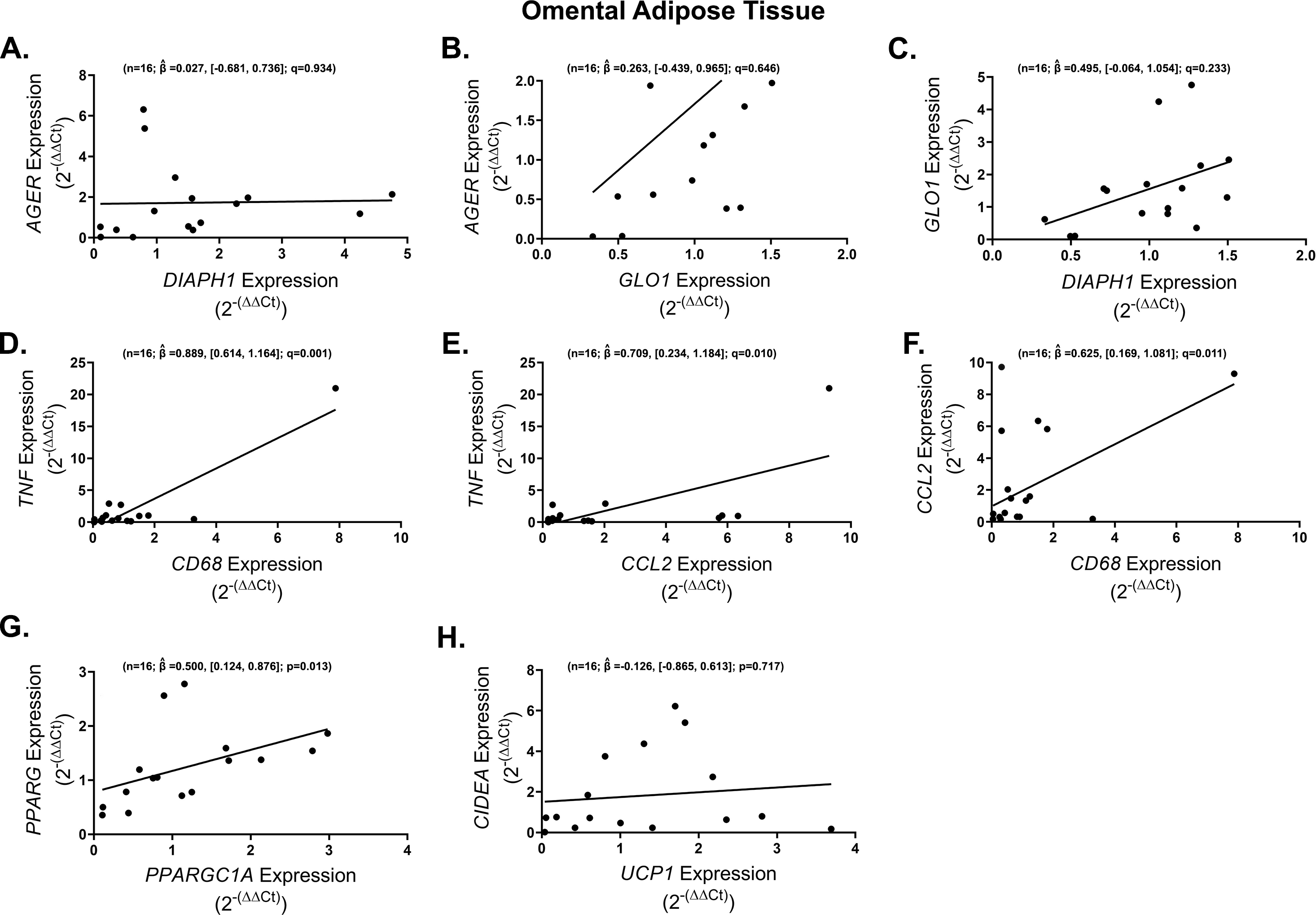

Supplement: Supplementary file 2 — Supplemental Figure 1 [file 41366_2021_878_MOESM2_ESM.tif]

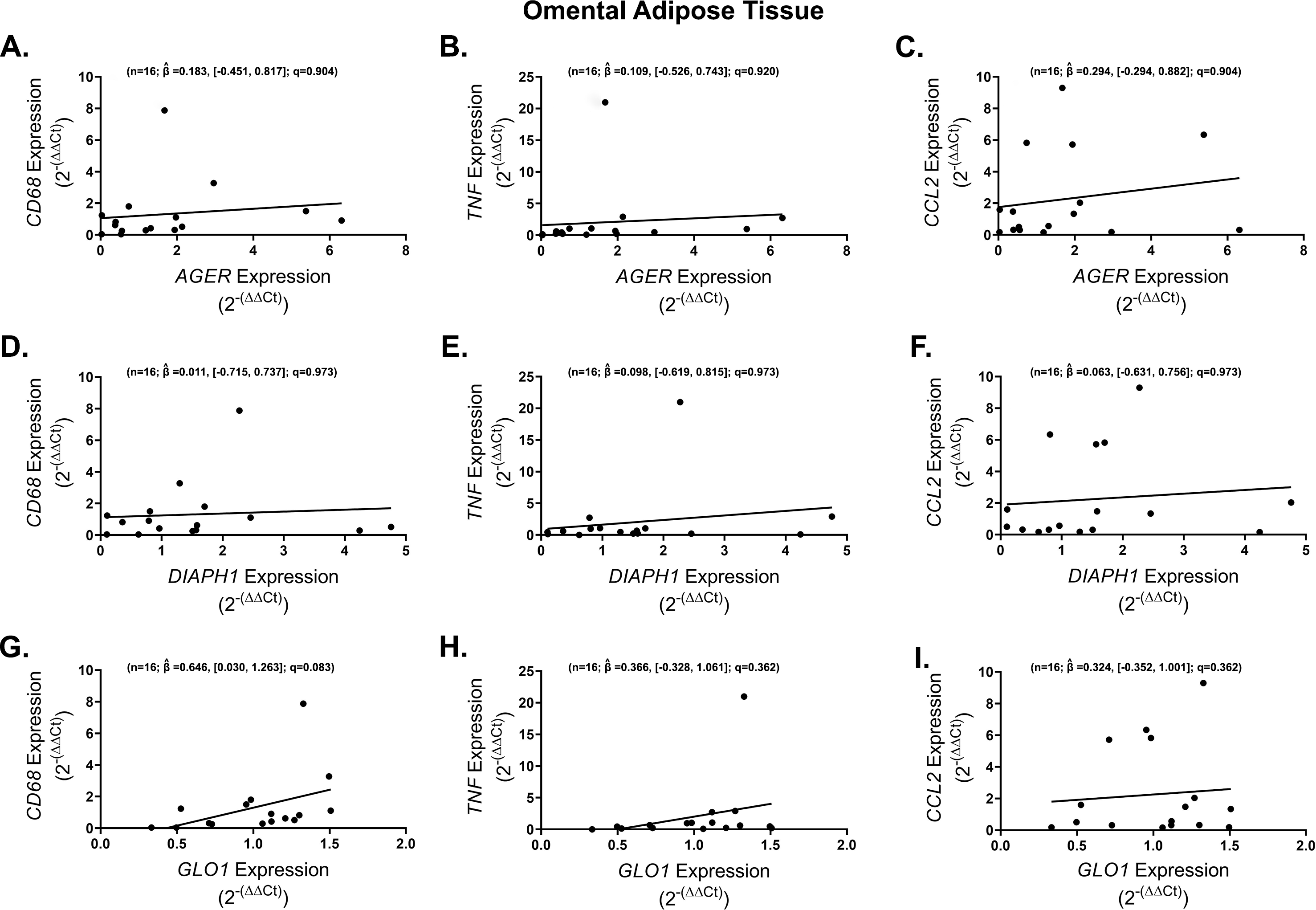

Supplement: Supplementary file 3 — Supplemental Figure 2 [file 41366_2021_878_MOESM3_ESM.tif]

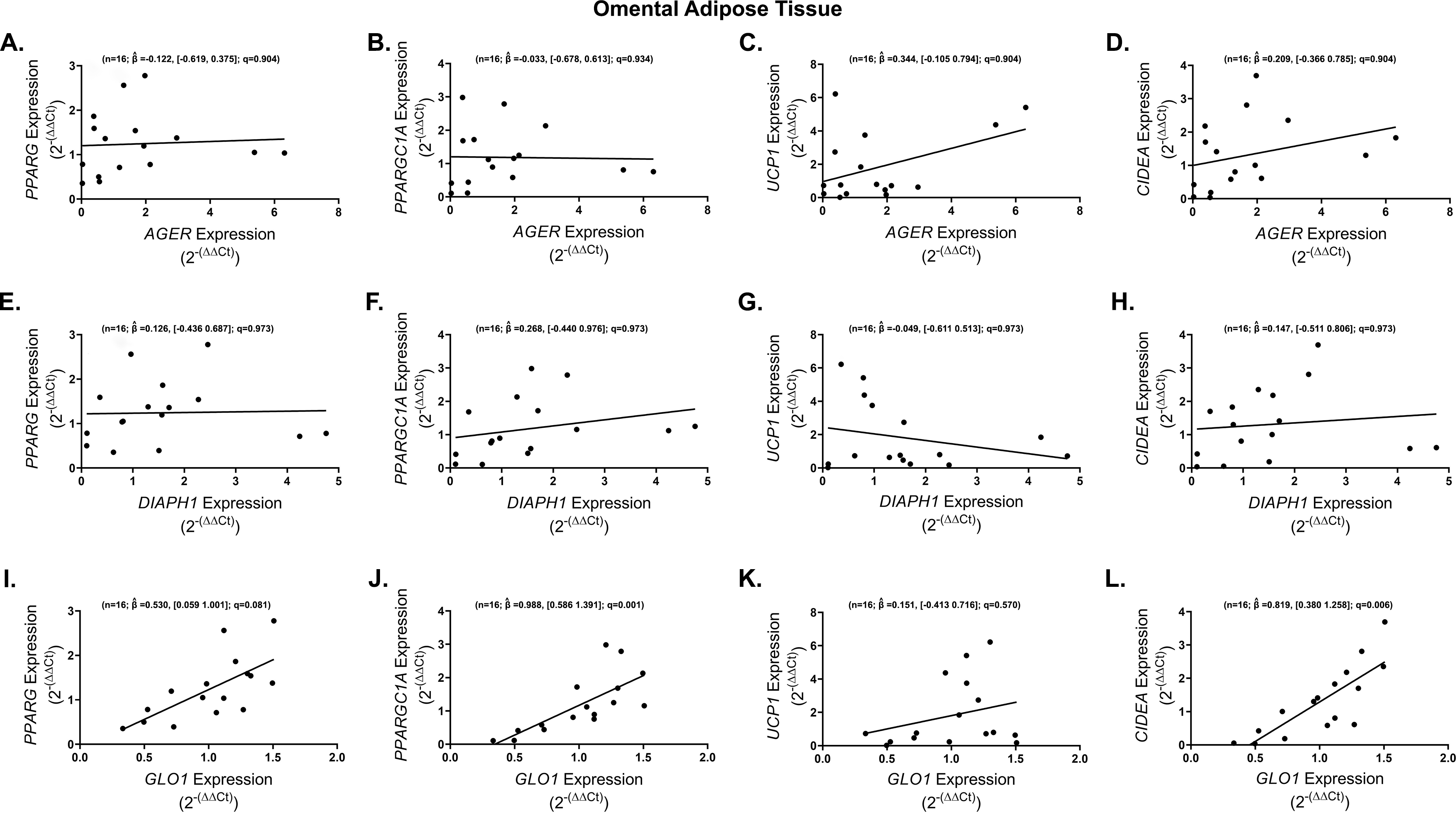

Supplement: Supplementary file 4 — Supplemental Figure 3 [file 41366_2021_878_MOESM4_ESM.tif]

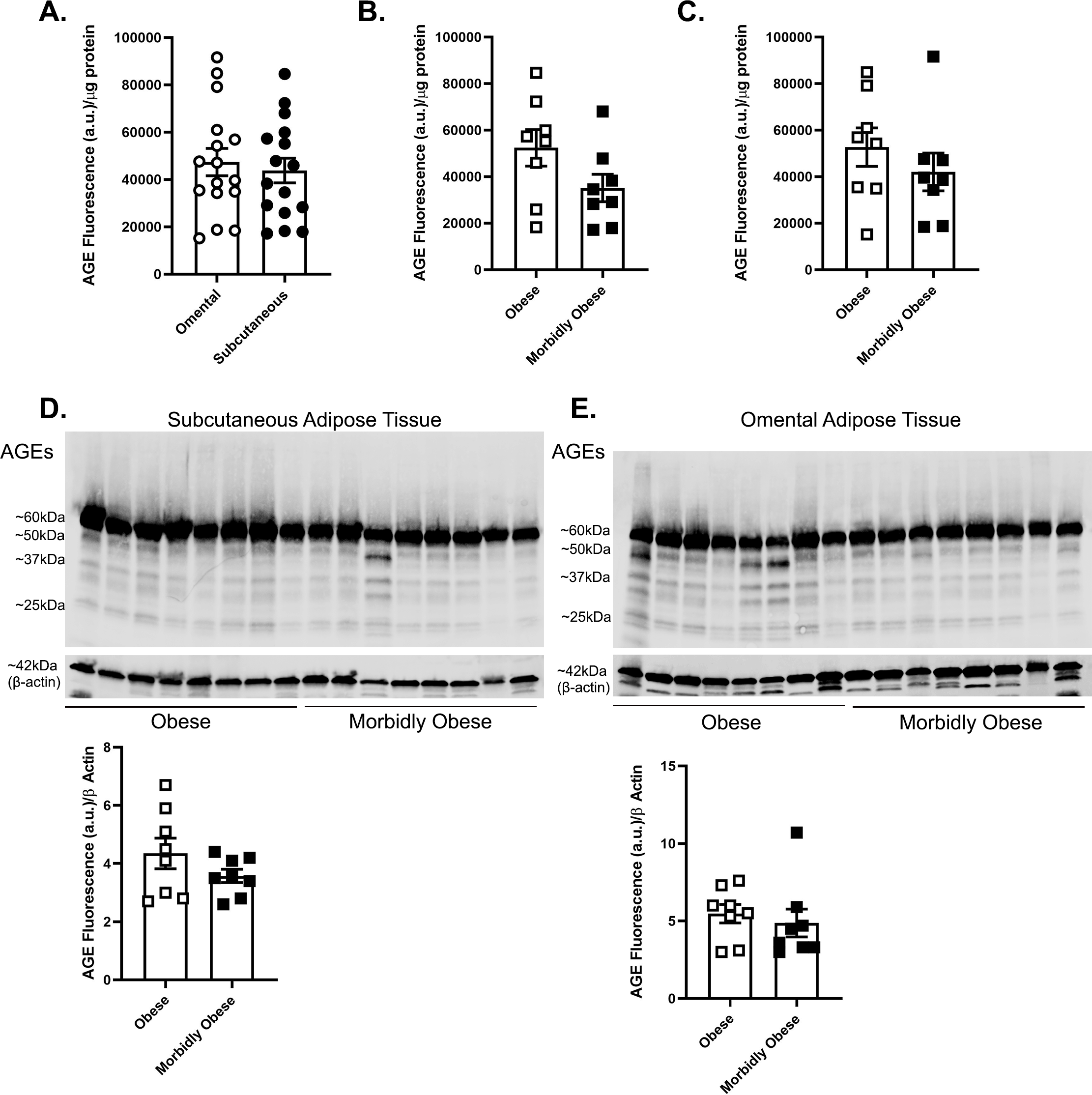

Supplement: Supplementary file 5 — Supplemental Figure 4 [file 41366_2021_878_MOESM5_ESM.tif]

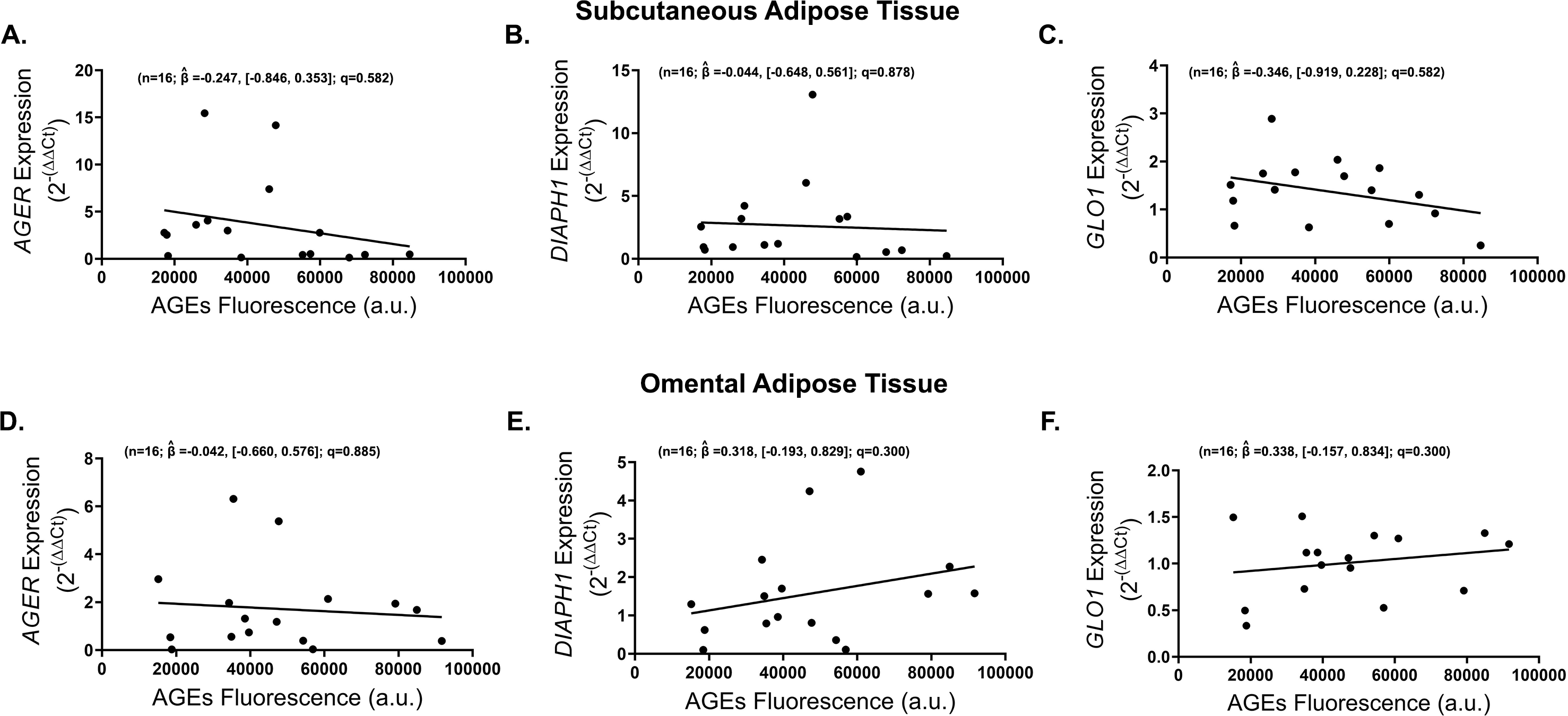

Supplement: Supplementary file 6 — Supplemental Figure 5 [file 41366_2021_878_MOESM6_ESM.tif]
